# The cutoff values of visceral fat area and waist circumference for identifying subjects at risk for metabolic syndrome in elderly Korean: Ansan Geriatric (AGE) cohort study

**DOI:** 10.1186/1471-2458-9-443

**Published:** 2009-12-02

**Authors:** Ji A Seo, Byoung Gwon Kim, Hyunjoo Cho, Hye Sook Kim, Juri Park, Sei Hyun Baik, Dong Seop Choi, Moon Ho Park, Sangmee Ahn Jo, Young Ho Koh, Changsu Han, Nan Hee Kim

**Affiliations:** 1 Division of Endocrinology and Metabolism, Department of Internal Medicine, Korea University Ansan Hospital, 516 Gojan-1-dong, Danwon-gu, Ansan, Gyeonggi-do 425-707, Republic of Korea; 2 Department of Occupational and Environmental Medicine, Korea University Ansan Hospital, 516 Gojan-1-dong, Danwon-gu, Ansan, Gyeonggi-do 425-707, Republic of Korea; 3 Department of Neurology, Korea University Ansan Hospital, 516 Gojan-1-dong, Danwon-gu, Ansan, Gyeonggi-do 425-707, Republic of Korea; 4 Center for Biomedical Sciences, Biomedical Research Center, National Institute of Health, 194 Tongil-lo, Eunpyung-gu, Seoul 122-701, Republic of Korea; 5 Department of Psychiatry, Korea University Ansan Hospital, 516 Gojan-1-dong, Danwon-gu, Ansan, Gyeonggi-do 425-707, Republic of Korea

## Abstract

**Background:**

In Korea, the cutoff values of waist circumference (WC) for the identification of metabolic syndrome (MetS) were suggested to be 90 cm for men and 85 cm for women based on the analysis mainly in middle-aged adults. As aging is associated with increased fat, especially abdominal visceral fat, the cutoff value of WC may differ according to age. In addition, the usefulness of visceral abdominal fat area (VFA) to predict MetS in the elderly has not been studied yet. We aimed to suggest WC and VFA criteria and to compare the predictability of WC and VFA to identify people at risk for MetS.

**Methods:**

A total of 689 elderly subjects aged ≥63 years (308 men, 381 women) were chosen in this cross-sectional study from an ongoing, prospective, population-based study, the Ansan Geriatric (AGE) cohort study. VFA was measured by single slice abdominal computed tomography scanning. The metabolic risk factors except WC (plasma glucose, blood pressure, serum triglycerides and HDL cholesterol levels) were defined using modified NCEP-ATP III criteria. We estimated the accuracy of VFA and WC for identifying at least two of these factors by receiver operating characteristic (ROC) curve analysis.

**Results:**

Two hundred three of 308 men and 280 of 381 women had ≥2 metabolic risk factors. The area under the ROC curve (AUC) value for VFA to predict the presence of ≥2 metabolic risk factors was not significantly different from that for WC (men, 0.735 and 0.750; women, 0.715 and 0.682; AUC values for VFA and WC, respectively). The optimal cutoff points for VFA and WC for predicting the presence of ≥2 metabolic risk factors were 92.6 cm^2 ^and 86.5 cm for men and 88.9 cm^2 ^and 86.5 cm for women.

**Conclusion:**

WC had comparable power with VFA to identify elderly people who are at risk for MetS. Elderly Korean men and women had very similar cutoff points for both VFA and WC measurements for estimating the risk of MetS. Age-specific cutoff point for WC might be considered to identify subjects at risk for MetS.

## Background

In 2006, the International Diabetes Federation proposed that central obesity is a prerequisite for the diagnosis of metabolic syndrome (MetS). Central obesity is defined using ethnicity-specific cutoff point of waist circumference (WC) [1]. The Korean Society for the Study of Obesity (KOSSO) set Korean-specific WC cutoff points of 90 cm for men and 85 cm for women [2]. These numbers were derived through analysis of data collected during the Korean National Health and Nutrition Examination Survey (KNHANES) 1998 [3], which is a representative data of non-institutionalized Korean civilians. However, the KOSSO did neither provide the cutoff point for visceral abdominal fat area (VFA) nor did differentiate WC criteria by age group.

Normal aging process is characterized by progressive increase in fat mass and more central distribution of adipose tissue [4,5]. These changes can have important consequences on the profile of risk factors for developing MetS. Women are particularly susceptible to increase in visceral fat as they go through menopause [6]. Considering that the accumulation of visceral fat with aging can be more prominent in women than in men, the gender difference in current Korean-specific criteria for abdominal obesity could be decreased with aging. Recently, a few studies have reported that VFA and WC cutoff points for the identification of subjects at risk for developing MetS differ by age, with older women experiencing higher VFA and WC cutoff points than younger women [7,8].

We estimated optimal values for VFA, WC and body mass index (BMI) cutoff points for defining central obesity by comparing the predictive validity of the metabolic risk factors other than WC in elderly Korean men and women in order to verify the adequacy of the current criteria of abdominal obesity in elderly Koreans.

## Methods

### Study participants

This cross-sectional study was conducted within the framework of the Ansan Geriatric (AGE) Study. The AGE study is an ongoing prospective population-based cohort study including subjects who are at least 60 years old and live in Ansan, a suburb of Seoul, Republic of Korea. A random sample of these elderly Koreans (n = 2,767) was assembled in 2002. The sampling protocol and design of the AGE study has been previously reported [9]. From this database, we constructed the 1^st ^wave sample of the AGE cohort study that was age- and sex-stratified [10,11]. Briefly, all subjects received a telephone call and a letter 3 times inviting them to attend a comprehensive health screening at the Geriatric Health Clinic and Research Institute, Korea University Hospital, Ansan, Korea. After excluding the subjects who refused to participate or who were not reached at this invitation, a total of 1,391 subjects were recruited between September 2004 and March 2006 and were regarded as the 1^st ^wave sample. The follow-up assessment (2^nd ^wave study) was performed two years later (25.6 ± 5.1 months) between April 2006, and January 2008. A total of 841 subjects (2^nd ^wave sample set) were followed from the 1^st ^wave sample with the same recruitment protocol of the 1^st ^wave sample. This study was conducted as a cross-sectional study from the 2^nd ^wave sample set of the AGE Study because abdominal computed tomography (CT) scan was taken from the 2^nd ^wave study. Among the 2^nd ^wave sample set (n = 841), a total of 152 subjects were excluded because they had insufficient clinical data (n = 12) or no available abdominal CT scan (n = 140).

A total of 689 Korean subjects (308 men, 381 women; mean age, 70.8 ± 5.0 years; range, 63-86 years) were ultimately eligible for participation in this analysis. Written informed consent was obtained from each individual. The study protocol was approved by the institutional review board of the AGE study.

### Anthropometric measurements and blood tests

Anthropometric measurements for each participant were taken after an overnight fast while the subject wore light clothing and no shoes. Height was determined using a fixed wall-scale measuring device and was measured to the nearest 0.1 cm. Weight was measured to the nearest 0.1 kg using an electronic scale that was calibrated prior to each measurement. BMI was calculated as the weight in kilograms divided by the square of the height in meters. The WC was measured twice to the nearest 0.5 cm in a horizontal plane at the level of the umbilicus at the end of normal expiration. If the variation between these two measurements was greater than 2 cm, a third measurement was taken and the mean was calculated by using the two closest measurements. Blood pressure was measured using a mercury sphygmomanometer (Baumanometer; W.A.Baum, Copiague, NY) in the supine position after at least a 10-min rest period by trained technicians. Three measurements were taken from all the subjects at 2-min intervals, and the average of the last two measurements was used. These were recorded to the nearest 2 mmHg. Blood samples were taken in the morning after an overnight fast. All subjects had a 75 g oral glucose tolerance test (OGTT). We measured fasting and post two hour OGTT-glucose levels. Plasma glucose, serum triglycerides and HDL-cholesterol levels were measured using an autoanalyzer (ADVIA1650; Siemens, NY, USA).

### Definition of the metabolic risk factors except WC

The metabolic risk factors were defined using the modified NCEP-ATP III criteria, with the exception of WC [12] as follows: 1) fasting plasma glucose ≥100 mg/dL or post two hour OGTT-glucose ≥ 140 mg/dL or taking medication for previously diagnosed type 2 diabetes; 2) blood pressure ≥ 130/85 mmHg or taking medication for previously diagnosed hypertension; 3) serum triglycerides levels ≥ 150 mg/dL; 4) serum HDL cholesterol levels < 40 mg/dL in men, < 50 mg/dL in women.

### Determination of visceral fat area using computed tomography

The abdominal adipose tissue areas were quantified by a single slice computed tomography (CT) scan at a 120 kV exposure (Brilliance 64; Philips, Cleveland, USA). A 5-mm CT slice scan was acquired at the L4-L5 vertebral interspace to measure the total abdominal and visceral abdominal fat areas (VFA) by measuring the mean value of all pixels within the range of -190 to -30 Hounsfield units. The images were converted into files compatible with a commercial software program (EBW; Philips, Cleveland, USA). Subcutaneous abdominal fat area (SFA) was determined by subtracting VFA from total abdominal fat area.

### 10-year Framingham coronary heart disease risk score

After excluding subjects with a previous history of cardiovascular disease (n = 38), 10-year Framingham coronary heart disease risk score (10-Yr FRS) was calculated as suggested by D'Agostino et al., which estimates risks for coronary heart disease in the next 10 years [13]. The equation includes risk variables such as age, diagnosis of diabetes, current smoking, categories of systolic/diastolic blood pressure, total cholesterol and HDL cholesterol. The 10-Yr FRS was evaluated as high risk for greater than 20%.

### Statistical analysis

Continuous variables were expressed as gender-specific means and standard deviations. Discrete variables were expressed as gender-specific proportions. For continuous variables, a Student's t-test was used to assess differences in means between the groups. For categorical data, a chi-square test was used to assess differences in proportions across the categories.

A receiver operating characteristic (ROC) curve analysis was used to assess the accuracy of VFA, WC, and BMI for identifying at least two of the metabolic risk factors, or for identifying each factor separately. ROC curve analysis for identifying a greater than 20% 10-Yr FRS was performed after excluding subjects with a previous history of cardiovascular disease (n = 38). A comparison of the diagnostic abilities for each test was performed using the area under the curves (AUC). The significance of the difference between the two areas was assessed using the method described by Hanley and McNeil [14,15]. The optimal cutoff points were obtained from the point on the ROC curve which was closest to (0, 1). This point was calculated as the minimum value of the square root of [(1-sensitivity)^2^+(1-specificity)^2^] [16]. We performed a simple regression analysis to define the correlations between VFA and WC. All reported *p *values were two-tailed. *P*-values of less than 0.05 were considered statistically significant. Statistical analyses were conducted using SAS version 9.1 for Windows (SAS Institute Inc., Cary, NC).

## Results

### Subject characteristics

Table 1 outlines the baseline characteristics of our subjects. The mean age of the subjects was about 70 years of age. The mean BMI was 24.0 kg/m^2 ^in men and 25.0 kg/m^2 ^in women. Men and women had very similar mean WCs of around 88 cm. SFA, but not VFA, was significantly greater in women than in men. The proportion of subjects who had two or more metabolic risk factors was 65.9% in men and 73.5% in women. The mean age of subjects who had two or more metabolic risk factors was not different from those with less than one risk factor. Both men and women with two or more metabolic risk factors were more obese and had worse metabolic characteristics than subjects with less than one risk factor. Women were more likely to have reduced HDL-C levels than men. After excluding subjects with a previous history of cardiovascular disease, the proportion of subjects with more than 20% of 10-Yr FRS was 66.4% in men and 28.7% in women. The upper 80^th ^percentile of VFA, WC, and BMI was 142.7 cm^2^, 94 cm, and 26.2 kg/m^2 ^for men and 145.4 cm^2^, 95 cm, and 27.4 kg/m^2 ^for women.

**Table 1:** Characteristics of study subjects according to the presence of two or more metabolic risk factors except WC in elderly Koreans

		Men			Women	
	
	Total	Two or more metabolic risk factors of the modified NCEP-ATP III of the criteria except WC		Total	Two or more metabolic risk factors of the modified NCEP-ATP III of the criteria except WC	
				
		Absent	Present		Absent	Present
N (%)	308	105 (34.1)	203 (65.9)	381	101 (26.5)	280 (73.5)
Age (years)	71.2 ± 5.2	70.6 ± 5.1	71.6 ± 5.3	70.5 ± 4.7	70.5 ± 5.0	70.5 ± 4.7
Body mass index (kg/m^2^)	24.0 ± 2.8	22.6 ± 2.4	24.7 ± 2.7*	25.0 ± 3.1^†^	23.4 ± 3.4	25.6 ± 2.8*
Waist circumference (cm)	88.0 ± 7.6	83.9 ± 7.0	90.2 ± 7.0*	88.5 ± 8.3	84.6 ± 9.1	90.0 ± 7.5*
VFA (cm^2^)	103.6 ± 49.1	78.3 ± 40.4	116.7 ± 48.2*	108.9 ± 45.4	84.0 ± 37.5	117.9 ± 44.7*
SFA (cm^2^)	157.1 ± 50.9	138.6 ± 47.5	166.7 ± 50.1*	239.4 ± 67.8^†^	215.5 ± 74.9	248.0 ± 63.0*
Fasting glucose (mg/dl)	96.5 ± 17.0	90.0 ± 8.9	99.8 ± 19.0*	95.8 ± 21.1	88.1 ± 10.6	98.5 ± 23.1*
post 2 hr OGTT-glucose (mg/dl)	129.5 ± 50.8	103.7 ± 34.0	145.5 ± 52.9*	138.5 ± 51.7	109.1 ± 27.2	150.9 ± 54.5
High glucose (n, (%))^a^	151 (49.0)	15 (14.3)	136 (67.0)*	187 (49.1)	10 (9.9)	177 (63.2)*
Taking oral hypoglycemic medication or insulin (n,(%))	41(13.3)	3 (2.9)	38 (18.7)*	44 (13.6)	3 (3.0)	41 (14.6)*
Systolic blood pressure	129.4 ± 15.6	123.2 ± 16.2	132.6 ± 14.3*	127.3 ± 16.1	120.0 ± 13.4	129.9 ± 16.2*
Diastolic blood pressure	76.1 ± 8.5	73.9 ± 7.5	77.3 ± 8.7*	75.2 ± 8.0	72.1 ± 8.1	76.3 ± 7.7*
High blood pressure (n, (%))^b^	205 (66.6)	35 (33.3)	170 (83.7)*	249 (65.4)	35 (34.7)	214 (76.4)*
Taking antihypertensive medication (n, (%))	140(45.5)	20 (19.1)	120 (59.2)*	185 (48.6)	21 (20.8)	164 (58.6)*
Triglycerides (mg/dl)	137.1 ± 80.9	97.9 ± 29.3	157.4 ± 91.0*	146.5 ± 80.9	92.2 ± 26.9	166.0 ± 84.9*
Hypertriglyceridemia (n, (%))^c^	96 (31.2)	5 (4.8)	91 (44.8)*	138 (36.2)	1 (1.0)	137 (48.9)*
HDL cholesterol (mg/dl)	41.0 ± 10.2	46.8 ± 9.4	38.1 ± 9.2*	43.1 ± 10.4^†^	51.6 ± 9.8	40.0 ± 8.8*
Low HDL cholesterol (n,(%))^d^	159 (51.6)	16 (15.2)	143 (70.4)*	286 (75.1)^†^	39 (38.6)	247 (88.2)*
Taking lipid-lowering medication (n, (%))	31 (10.1)	8 (7.6)	23 (11.3)	44 (11.6)	10 (9.9)	34 (12.1)

Data are mean ± S.D. or %. VFA, Visceral abdominal fat area; SFA, Subcutaneous abdominal fat area

^a^High glucose was diagnosed if fasting glucose ≥100 mg/dl or post 2 hr OGTT-glucose ≥140 mg/dl or the subjects with oral hypoglycemic medication or insulin.

^b^High blood pressure was diagnosed if systolic blood pressure was ≥130 mmHg, diastolic blood pressure ≥85 mmHg, or the subjects with antihypertensive medications.

^c^Hypertriglyceridemia was diagnosed if triglycerides was ≥150 mg/dl in men and women.

^d^Low HDL cholesterol was diagnosed if HDL cholesterol was < 40 mg/dl in men and < 50 mg/dl in women.

*: *p *<0.01 compared with subjects without two or more metabolic risk factors in each gender.

^†^: p < 0.01 compared with men.

### Predictability and the cutoff points of VFA, WC, and BMI for identifying the presence of two or more metabolic risk factors, or a greater than 20% 10-Yr FRS

**Figure 1:**
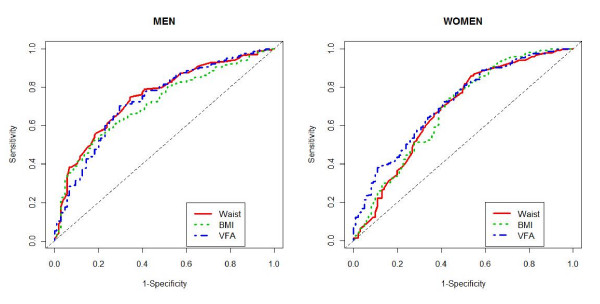
**ROC curves to identify ≥2 metabolic risk factors other than WC**. In both men and women, the AUC values for VFA did not show higher values compared to WC and BMI.

AUC values of VFA, WC, and BMI for identifying the presence of two or more metabolic risk factors are presented in Table 2. In both men and women, the AUC for VFA did not show higher values compared to WC and BMI (Table 2, Fig. 1). VFA had greater AUC values compared to WC or BMI only in distinguishing women with hypertriglyceridemia or men with low HDL cholesterol levels.

**Table 2:** Areas under the ROC curve of VFA, WC and BMI to identify the presence of the metabolic risk factors other than WC

	Men (n = 308)		Women (n = 381)	
	
	ROC curve area (95% CI)	*P *value compared with VFA	ROC curve area (95% CI)	*P *value compared with VFA
Two or more metabolic risk factors of the modified NCEP-ATP III criteria other than waist circumference				
VFA	0.735 (0.676-0.794)		0.715 (0.656-0.774)	
WC	0.750 (0.693-0.807)	0.509	0.682 (0.617-0.747)	0.229
BMI	0.717 (0.659-0.775)	0.512	0.684 (0.619-0.749)	0.270
				
Fasting glucose ≥ 100 mg/dl or post 2 hr OGTT-glucose ≥ 140 mg/dl or medication for diabetes				
VFA	0.670 (0.609-0.731)		0.624 (0.568-0.680)	
WC	0.684 (0.624-0.744)	0.431	0.612 (0.556-0.668)	0.687
BMI	0.637 (0.575-0.699)	0.283	0.581 (0.524-0.638)	0.100
				
High blood pressure ^a^				
VFA	0.653 (0.588-0.718)		0.614 (0.554-0.674)	
WC	0.671 (0.609-0.733)	0.445	0.608 (0.547-0.669)	0.842
BMI	0.676 (0.613-0.739)	0.397	0.617 (0.556-0.678)	0.898
				
Hypertriglyceridemia ^b^				
VFA	0.647 (0.585-0.709)		0.673 (0.618-0.728)	
WC	0.622 (0.555-0.689)	0.319	0.615 (0.558-0.672)	0.028
BMI	0.600 (0.532-0.668)	0.080	0.615 (0.559-0.671)	0.032
				
Low HDL cholesterol ^c^				
VFA	0.692 (0.633-0.751)		0.620 (0.553-0.687)	
WC	0.664 (0.604-0.724)	0.235	0.597 (0.526-0.668)	0.431
BMI	0.631 (0.569-0.693)	0.018	0.585 (0.514-0.656)	0.229

VFA, visceral fat area; WC, waist circumference; BMI, body mass index

^a^High blood pressure was diagnosed if systolic blood pressure was ≥130 mmHg, diastolic blood pressure ≥85 mmHg, or the subjects was receiving antihypertensive medications.

^b^Hypertriglyceridemia was diagnosed if triglycerides was ≥ 150 mg/dl in men and women.

^c^Low HDL cholesterol was diagnosed if HDL cholesterol was < 40 mg/dl in men and < 50 mg/dl in women.

The optimal cutoff points of VFA for identifying the presence of two or more metabolic risk factors were 92.6 cm^2 ^for men and 88.9 cm^2 ^for women (Table 3). For WC, the optimal cutoff point was the same for both men and women (86.5 cm). This cutoff point identified 75% of men who had increased metabolic risk, while the KOSSO's cutoff point of 90 cm identified only 54%. For BMI, the optimal cutoff points were 23.7 kg/m^2 ^for men and 23.9 kg/m^2 ^for women. All of the cutoff points were very similar between men and women. In addition, both genders had similar optimal VFA and WC cutoff points for identifying ≥20% 10-Yr FRS (VFA and WC; 102.4 cm^2 ^and 87.0 cm in men; 105.9 cm^2 ^and 88.7 cm in women). The AUC for VFA did not show higher values compared to WC for identifying ≥20% 10-Yr FRS (AUC values of VFA and WC; 0.690 and 0.650 in men (p = 0.11); 0.642 and 0.640 in women (p = 0.96)). The optimal VFA and WC cutoff points did not change when we excluded individuals taking oral hypoglycemic medication, insulin, or lipid-lowering medication (VFA and WC; 92.6 cm2 and 86.5 cm in men (n = 223); 88.9 cm2 and 85.5 cm in women (n = 270)).

**Table 3:** The optimal cutoff points of VFA, WC, and BMI to detect subjects with two or more metabolic risk factors except WC compared to various criteria

		Cut point	Sensitivity (%)	Specificity (%)	PPV (%)	NPV (%)	**J value**^**a**^	Distance
Men (n = 308)								
VFA(cm^2^)	Optimal cut point ^a^	92.6	70.4	70.5	82.2	44.8	0.41	0.42
WC (cm)	Optimal cut point ^a^	86.5	74.9	65.7	80.9	42.5	0.41	0.43
	KOSSO's cut point ^b^	90.0	54.2	81.9	85.3	52.0	0.36	0.49
BMI (kg/m^2^)	Optimal cut point ^a^	23.7	63.1	70.5	80.5	50.3	0.34	0.47
	WHO's cut point ^c^	25.0	45.8	84.8	85.3	55.3	0.31	0.56
Women (n = 381)								
VFA (cm^2^)	Optimal cut point ^a^	88.9	72.5	59.4	83.2	56.2	0.32	0.49
WC (cm)	Optimal cut point ^a^	86.5	69.3	60.4	82.9	58.5	0.30	0.50
	KOSSO's cut point ^b^	85.0	76.4	51.5	81.4	55.9	0.28	0.54
BMI (kg/m^2^)	Optimal cut point ^a^	23.9	72.5	58.4	82.9	56.6	0.31	0.50
	WHO's cut point ^c^	25.0	54.6	64.4	81.0	66.2	0.19	0.58

PPV, positive predictive value; NPV, negative predictive value

^a ^: Optimal cut point from the present study for abdominal obesity

^b^: KOSSO's criteria for abdominal obesity [2]

^c ^: WHO-Asia Pacific Region criteria for obesity [23]

### Gender-specific WC values corresponding to VFA

When we performed the simple regression analyses between VFA and WC, the regression lines indicated that a VFA of 92.6 cm^2 ^(cutoff point from the ROC analysis) corresponded to a WC of 86.8 cm in men. In women, a VFA of 88.9 cm^2 ^(cutoff point from the ROC analysis) corresponded to a WC of 86.2 cm. Therefore, men and women had very similar predicted values for WC based on the cutoff points of VFA.

### Prevalence of MetS using modified NCEP-ATP III criteria

**Figure 2:**
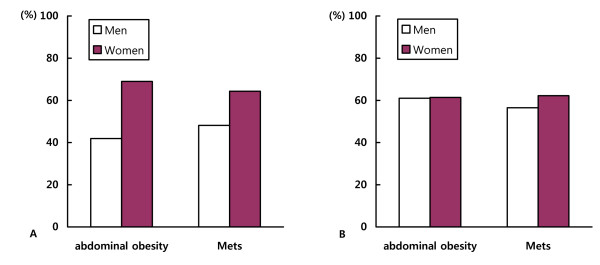
**The proportion of subjects with abdominal obesity and metabolic syndrome using modified NCEP-ATP III criteria with KOSSO's WC cutoff points (≥ 90 cm for men and ≥ 85 cm for women) (A) or with our WC cutoff points (≥86.5 cm for both men and women) (B)**.

When we used the modified NCEP-ATP III criteria with KOSSO's cutoff point for abdominal obesity (≥90 cm for men and ≥85 cm for women) [2], the prevalence of abdominal obesity and MetS was consistently higher in women than in men (*p *< 0.001 between genders, Fig 2A). In contrast, the prevalence of abdominal obesity and MetS using WC cutoff points suggested in this study (≥86.5 cm for both men and women) was similar in both genders (*p *> 0.05 between genders, Fig. 2B).

## Discussion

We demonstrated that the optimal WC cutoff points for predicting two or more metabolic risk factors or for identifying a greater than 20% 10-Yr FRS were same in elderly Korean men and women. Optimal VFA and BMI cutoff points were also similar in both genders. Given our findings, WC cutoff points determined for young and middle-aged adults may not appropriately characterize elderly Koreans.

In 2006, KOSSO (the Korean Society for the Study of Obesity) set WC criteria for central obesity at 90 cm for men and 85 cm for women. These numbers are based on the upper 80th percentile for WC values in middle-aged Koreans [2]. In the present study, we found that the WC cutoff point was similar in both genders when using the 80^th ^percentile of WC. This is because the mean WC of men was not different from that of women. Young men typically have a higher WC compared to women. Although there are some ethnic differences [17,18], these differences become smaller with age. In our elderly subjects, women had similar mean WC and even slightly higher BMI compared to men. We confirmed that the difference of mean WC between genders decreased with age in the Third KNHANES, a nationwide survey which was conducted in 2005 (mean WC in subjects with 20-63 years old (n = 6480), 76.3 cm in men and 72.0 cm in women; vs. in subjects ≥63 years (n = 1069), 85.0 cm in men and 83.2 cm in women, unpublished data). Another study in Chinese population demonstrated the same feature. Younger Chinese men had higher mean WC and BMI than younger Chinese women but older Chinese men had comparable or even lower mean WC and BMI than older Chinese women [19]. When comparing the present study and the recent publication with 816 middle-aged Korean subjects who underwent routine health examinations [20], appropriate VFA cutoff points for middle-aged men in their study was comparable to those for elderly men in this study (100 cm^2 ^vs. 93 cm^2^), whereas cutoff points for young women was lower than those for elderly women in this study (70 cm^2 ^vs. 89 cm^2^). It could be from that women have a relatively greater increase in visceral fat after menopause [21]. Changes in sex hormones, dietary intake, and energy expenditure during menopause can be related to increase in visceral fat and prevalence of obesity in senescence [6,22]. Considering these changes in body composition and metabolism in elderly women, the cardiovascular risk associated with central obesity may be different from that of younger women. Some Asian reports have indicated that the WC cutoff points for a middle aged population are different from those of older population [8,19]. It should be further investigated whether age-related changing pattern of obesity is specific to Asians.

The prevalence of MetS in men was greater than in women until the fifth decade in KNHANES 1998 [3], using the definition of abdominal obesity recommended by the WHO Western Pacific Region of WC ≥90 cm in men and ≥80 cm in women [23,24]. However, after that period, women had a significantly higher prevalence of MetS than men. These results may be partially explained by relatively lower cutoff point of WC for women. When we used higher WC cutoff point for women such as the KOSSO criteria for abdominal obesity (WC ≥90 cm in men, ≥85 cm in women), the prevalence of MetS was still higher in women (48.1% in men and 64.3% in women). However, it was not different according to gender with the optimal WC cutoff points that we identified (61.0% in men and 61.4% in women). These results indicate that age-specific WC criteria which may reflect age-associated body compositional change will more consistently identify high risk subjects, especially in old age population compared to the uniform criteria. However, this feature is not evident in certain populations. In the Third National Health and Nutrition Examination Survey III (NHANES III) of American adults, the prevalence of MetS was similar between genders throughout all age groups despite using the same cutoff points for central obesity in both young and old populations [25]. This could be due to continued differences in the mean WC between men and women throughout their entire lifespan in certain ethnics [26].

The predictability of WC for identifying the risk of MetS was comparable to that of VFA in our study. Some studies in Japanese subjects reported that VFA was better than WC and BMI for identification of subjects with two or more components of MetS [8,27]. In contrast, a Korean study in obese middle-aged women showed that the VFA is not superior to WC for predicting metabolic risk factors [28]. Another Japanese study has shown that VFA does not have better correlation with carotid intima-media thickness as a surrogate measurement of atherosclerosis than waist-hip-ratio or WC [29]. It should be investigated in the future whether VFA is really superior to WC for predicting the clustering of metabolic risk factors in general population. Because WC is an inexpensive and clinically feasible measurement compared to the direct imaging required for assessing visceral fat, we suggest that WC measurements are sufficient for the detection of central obesity in correlation with the risk of MetS in elderly Koreans.

Although the present study is the first population-based cohort report in Korea that addresses the VFA cutoff point, it has some limitations. Firstly, we only showed the cross-sectional association between WC, VFA and MetS. Further investigation with prospective design is needed to clarify more reliable cutoff points of WC and VFA, above which the development of cardiovascular diseases increase. Secondly, we cannot provide comparative data between young and elderly subjects to estimate different WC and VFA criteria according to changes in body composition. However, the present study may reliably present metabolic features in elderly due to relatively large number of elderly subjects.

## Conclusion

According to both VFA and WC measurements, elderly Korean men and women had very similar cutoff points for the risk of MetS. WC and VFA were similarly useful for identifying people with metabolic risk factors in elderly Korean subjects. We propose that age-specific optimal cutoff points for WC might be considered for identifying subjects at metabolic risks.

## Abbreviations

MetS: metabolic syndrome; WC: waist circumference; KOSSO: the Korean Society for the Study of Obesity; KNHANES: the Korean National Health and Nutrition Examination Survey; VFA: visceral abdominal fat area; BMI: body mass index; AGE study: the Ansan Geriatric cohort study; OGTT: oral glucose tolerance test; SFA: subcutaneous abdominal fat area; 10-Yr FRS: 10-year Framingham coronary heart disease risk score; ROC curve: receiver operating characteristic curve; AUC: area under the curve.

## Competing interests

The authors declare that they have no competing interests.

## Authors' contributions

The first author, JS designed this study, interpreted the data and wrote this manuscript. BK contributed to the analysis. HC contributed to the analysis. HK contributed to the analysis. JP contributed to the analysis, interpretation of data. SB contributed to the discussion. DC contributed to the discussion. MP contributed to the discussion and edited the manuscript. CH contributed to the discussion and edited the manuscript. SJ contributed to design the cohort, collect the data and edit the manuscript. YK contributed to design the cohort, collect the data and edit the manuscript. NK guided the study design, analysis, interpretation of data, discussion, and revised the manuscript critically. All authors read and approved the final manuscript.

## Pre-publication history

The pre-publication history for this paper can be accessed here:

http://www.biomedcentral.com/1471-2458/9/443/prepub
